# Zisha Ceramics Modulate Metal-Ion Cycling and Volatile Aroma Evolution During Sauce-Flavor Baijiu Aging

**DOI:** 10.3390/foods15142477

**Published:** 2026-07-13

**Authors:** Ben Ma, Xinjun Hu, Rui Zhang, Jiawei Li, Long You, Jianping Tian, Manjiao Chen, Haili Yang, Liangliang Xie, Huibo Luo, Dan Huang, Lei Zheng

**Affiliations:** 1 School of Mechanical Engineering, Sichuan University of Science and Engineering, Yibin 644000, China; 2 Key Laboratory of Brewing Biotechnology and Application of Sichuan Province, Yibin 644000, China; 3 Luzhou Laojiao Co., Ltd., Luzhou 646000, China; 18615738628@163.com (J.L.);; 4 National Engineering Research Center of Solid-State Brewing, Luzhou 646000, China

**Keywords:** Baijiu aging, Zisha ceramics, pore structure, metal ions, flavor compounds

## Abstract

Ageing vessels are critical to the sensory maturation of Baijiu, yet the interfacial material processes by which traditional ceramics modulate liquor chemistry remain insufficiently defined. Here, Zisha and ceramic-clay particles with distinct aluminosilicate frameworks, mineral compositions, and pore structures were investigated to elucidate their roles in sauce-flavor Baijiu ageing. Ceramic characterization was combined with elemental and volatile analyses using ICP-MS, GC-MS, and GC-IMS. The results showed that pore development in Zisha was primarily governed by fluxing metal oxides and the Si/Al framework, whereas metal-ion migration into Baijiu was regulated by the SiO_2_-rich matrix, pore accessibility, and trace elemental composition. Metal elements exhibited reversible interfacial exchange rather than simple leaching, revealing a dynamic metal-ion cycling process during ageing. This process was associated with selective remodeling of the volatile profile. Compared with ceramic-clay particles, Zisha promoted the accumulation of acetophenone, 3-pentanone, and pyrazine derivatives while reducing dimethyl disulfide and heptanal. Sensory evaluation of Baijiu aged in ceramic jars further validated these findings. These findings identify Zisha ceramics as active material–flavour interfaces and provide a mechanistic basis for the rational design of ceramic ageing vessels to direct Baijiu flavour maturation.

## 1. Introduction

As one of the world’s six major distilled spirits, Chinese Baijiu embodies profound cultural heritage and culinary traditions; its distinctive flavor complexity and sensory attributes have continuously evolved through thousands of years of brewing practice [1]. The production of Chinese Baijiu involves a series of processes, including raw material selection, soaking, cooking, saccharification and fermentation, solid-state distillation, aging, and blending, generating a chemically complex distilled spirit enriched in esters, acids, alcohols, carbonyls and heterocyclic compounds [2]. Ageing is a decisive post-distillation step in which freshly distilled Baijiu undergoes volatilization, esterification, hydrolysis, oxidation–reduction and intermolecular association, thereby reducing harsh and pungent notes while improving aroma integration, mouthfeel and sensory persistence [3]. Among the major aroma types of baijiu, sauce-flavor baijiu typically requires a longer ageing period, as its sensory characteristics depend on the gradual formation of complex notes such as sauce-like, roasted, acidic and aged aromas, rather than on the prominence of a single ester-driven fragrance [4,5]. During prolonged storage, the concentrations and ratios of esters, acids, aldehydes, ketones, pyrazines and sulfur-containing compounds are continuously reshaped, making ageing time and storage environment particularly critical for the development of its distinctive flavour profile [6].

Ceramic vessels have therefore remained central to Baijiu aging, especially for high-value liquors, because their inorganic matrices provide a chemically active boundary between the spirit, oxygen and the container wall [7]. Unlike stainless steel, ceramic bodies contain interconnected micro- and mesopores and leachable metal oxides that can regulate oxygen diffusion, adsorb or desorb trace aroma molecules, remove irritant low-boiling compounds and supply catalytic ions that redirect the evolution of volatile compounds [8]. Zisha ceramics represent a particularly important yet insufficiently understood class of ceramic materials for Baijiu ageing. Derived from mineral-rich clay resources, Zisha possesses a stable aluminosilicate framework, iron-bearing phases and a hierarchical pore architecture that distinguishes it from conventional pottery and porcelain [9]. These structural and compositional features have long been associated with its performance in tea brewing, where Zisha vessels are valued for their ability to modulate aroma release, taste perception and thermal behaviour [10]. In Baijiu aging, however, the specific functions of Zisha remain experimentally unexplored. Based on its unique material properties, we hypothesize that Zisha ceramics can serve as a highly active material–flavor interface to modulate liquor aging. Specifically, its distinctive pores are hypothesized to selectively adsorb or release volatile compounds, regulate the local oxygen microenvironment, and provide abundant interfacial sites for flavor transformation [11]. Concurrently, we postulate that metal oxides embedded within the ceramic matrix may gradually release ions such as Fe, Mn, Mg, K and Na into the ethanol–water system, where they can participate in redox reactions, complexation processes and catalytic pathways, thereby influencing the formation or degradation of aroma compounds [12,13]. Consequently, a critical knowledge gap remains: the interrelationships among Zisha mineral composition, sintering-induced pore structure, metal-ion migration, and volatile flavor evolution have not yet been systematically elucidated.

This study aimed to elucidate the specific physicochemical mechanisms by which Zisha ceramics modulate Baijiu flavor evolution compared with conventional ceramic materials. We hypothesized that the unique pore architecture and trace metal composition of Zisha matrices initiate a dynamic metal-ion cycling effect at the ceramic–liquor interface. This dynamic process is proposed to regulate the evolution of specific volatile compounds through selective adsorption and catalytic pathways. By integrating material characterization with food flavor chemistry, this work systematically links the structural and compositional properties of ceramic vessels to their precise mechanistic roles in flavor remodeling during the aging process.

## 2. Materials and Methods

### 2.1. Zisha Materials

To investigate the differences between Zisha and ceramic clay, two types of Zisha and a ceramic clay were selected as experimental raw materials. One of the Zisha raw materials was sourced from Jiangsu Province, whereas another Zisha material originated from Sichuan Province. The ceramic clay material was obtained from Sichuan Province. The ceramic clay raw materials used for ceramic production were designated as Jiangsu Zisha (YX), Sichuan Zisha (RX), and Sichuan ceramic clay (SL).

### 2.2. Particle Preparation

The raw materials were first sieved, ground, and pulped. They were milled into powder using a disk grinder and passed through a 60-mesh sieve. The powder was then mixed with 15% water, pressed into pellets, and subjected to drying and sintering.

The sintering procedure of the green bodies is illustrated in Figure S1. The unfired samples were dried in a vacuum oven at 45 °C for 24 h and then fired in a muffle furnace. The temperature was increased from room temperature to 300 °C at a rate of 3 °C/min and held for 60 min, followed by heating from 300 °C to 700 °C at a rate of 7 °C/min and holding for 60 min. Subsequently, the temperature was raised from 700 °C to 1200 °C at a rate of 5 °C/min and held for 60 min, and finally cooled from 1200 °C to room temperature at a rate of 5 °C/min.

The sintered samples were ground into small particles using a disk grinder and then passed through 20 and 40 mesh sieves. Particles in the 20–40 mesh range were collected to increase the contact area and accelerate the reaction rate. These particles were then ultrasonically cleaned and dried for further use in subsequent Baijiu storage experiments.

### 2.3. Material Characterization

The chemical composition of the samples was characterized using an X-ray fluorescence (XRF; Rigaku ZSX Primus III+, Tokyo, Japan) spectroscope. The crystalline phases were determined using an X-ray diffractometer (XRD, Rigaku Ultima IV, Tokyo, Japan) with Cu Kα radiation (λ = 1.5406 A). The thermal behaviors of various beneficial components were analyzed using a simultaneous thermogravimetric–differential scanning calorimeter (TG-DSC; Netzsch STA 449 F3, Selb, Germany). The morphology and microstructure of the samples were examined by field-emission scanning electron microscopy (SU8010; Hitachi, Tokyo, Japan). The porosity of the particles was measured using a high-performance automatic mercury porosimeter (Micromeritics AutoPore V 9620; Norcross, GA, USA). The reduction sites of the particles were investigated via temperature-programmed reduction (TPR; Microtrac BELCAT II, Tokyo, Japan). The valence states of metal elements in the materials were analyzed using an X-ray photoelectron spectroscope (XPS; Thermo Fisher K-Alpha, Waltham, MA, USA).

### 2.4. Baijiu Samples

The sauce-flavor Baijiu used in the experiments was obtained from Guoli Distillery, Sichuan Province. Four grams of ceramic particles were added to a 50 mL amber glass vial, followed by 40 mL of Baijiu, and the vial was sealed and stored in an oven at 45 °C. A destructive sampling approach was employed, wherein three independent parallel samples were prepared for each specific time point. Once opened, the samples were analyzed in their entirety to strictly preclude any headspace variations from interfering with the aging process. The Baijiu samples were designated as Jiangsu Zisha (YXx), Sichuan Zisha (RXx), Sichuan ceramic clay (SLx), and blank control (KBx), where x indicates the storage week (1, 2, 3, 4, 5, 6, 7, 8, and 9 weeks); 10 represented a storage period of 3 months.

### 2.5. Baijiu Analysis Methods

Trace volatile components were qualitatively and semi-quantitatively analyzed using gas chromatography–mass spectrometry to comprehensively evaluate the effects of ceramic particles on the formation of complex aroma layers and flavor profiles in Baijiu. The concentrations of leached metal ions were determined by inductively coupled plasma mass spectrometry. Gas chromatography–ion mobility spectrometry was employed for direct and rapid characterization of the complete volatile organic compound fingerprints of Baijiu, thereby enabling visualization of the differences in overall flavor profiles. All instrumental measurements (GC-MS, GC-IMS, and ICP-MS) were performed in independent biological triplicates (*n* = 3) to ensure analytical reliability, with data variability expressed as the mean ± standard deviation (SD). Detailed analytical parameters are provided in the Supplementary Materials.

### 2.6. Sensory Evaluation During Long-Term Natural Aging

To validate the effectiveness of the 45 °C isothermal accelerated aging experiment, a supplementary long-term natural storage study was conducted. The YX, RX, and SL materials were first fabricated into ceramic particles. Equal volumes of Baijiu samples were then sealed within traditional ceramic vat containing the YX, RX, and SL particles, respectively, alongside a control jar without particles. These samples were stored under natural room temperature and ambient humidity conditions for a period of 9 months.

Sampling was performed at four critical time points during the natural aging process: 1.5, 3, 6, and 9 months. A panel comprising five professionally trained tasters conducted blind sensory evaluations. To quantitatively assess the dynamic evolution of the Baijiu’s sensory qualities under long-term natural aging without relying on aggregate scoring, the samples from different storage periods were scored across specific, independent indicators. These dimensions included the raw liquor sensation, grain aroma, sauce aroma, floral and fruity notes, and sourness, as well as sweetness and aftertaste length.

## 3. Results and Discussion

### 3.1. Chemical Composition and Phase Analysis of Zisha

XRF spectroscopy was first used to characterize the YX, RX, and SL raw materials. The chemical compositions of YX, RX, and SL are summarized in Table S1. The major chemical constituents of all three materials were SiO_2_, Al_2_O_3_, and Fe_2_O_3_, despite variations in their relative concentrations. Moreover, trace metal oxides such as MgO, CaO, Na_2_O, and K_2_O were detected. SiO_2_and Al_2_O_3_ serve as the framework components of ceramics. Also, their concentration and ratio directly influence the refractory properties and sintering performance of the green bodies. Among the samples, RX exhibited the highest SiO_2_ concentration, conferring superior refractory performance. Zisha, as a natural composite clay, is rich in Fe_2_O_3_. As shown in Table S1, Zisha contains relatively high Fe_2_O_3_ concentrations, with YX having the highest iron concentration.

The chemical compositions of YX, RX, and SL specimens after sintering at 1200 °C are shown in Table 1. After sintering, the concentrations of oxides in all raw materials underwent certain changes, although the overall trends remained largely consistent with those observed in the raw material stage. Differences in the initial compositions of the raw materials impact the transformation of mineral phases and the formation of liquid phases during sintering, leading to variations in the chemical composition of the sintered bodies. Alkali and alkaline earth metal oxides act as strong fluxing agents in the ceramic bodies. They lower the melting temperature of the primary components and promote the formation of high-temperature liquid phases, thereby facilitating densification of the green bodies [14]. The total concentration of alkali and alkaline earth metal oxides (MgO + CaO + Na_2_O + K_2_O) in YX, RX, and SL was 4.23%, 2.87%, and 4.93%, respectively, indicating that RX contained significantly lower concentrations than YX and SL. The Fe_2_O_3_ concentration in both Zisha materials was markedly higher than that in SL, reflecting a greater enrichment of iron. Regarding the major chemical components, the differences among the three ceramic materials were mainly characterized by the higher SiO_2_ concentration in RX and the lower Fe_2_O_3_ concentration in SL. An excess of SiO_2_ can lead to surface silicification, thereby reducing the leaching capacity of metal elements. For trace components, the K_2_O concentration in RX (1.77%) was relatively low. Trace oxides such as K_2_O and TiO_2_ in ceramic materials can synergistically form surface acidic sites during Baijiu storage, promoting metal ion leaching and participating in the transformation of Baijiu flavor compounds.

Figure S2 presents the XRD patterns of YX, RX, and SL before and after sintering. The corresponding Rietveld refinement results are summarized in Table 2. The mineral assemblages of the three raw materials were similar prior to sintering, primarily comprising quartz, kaolinite, muscovite, hematite, and an amorphous phase. After sintering, the mineral assemblages still included quartz, mullite, hematite, and the amorphous phase. However, the composition of the crystalline phases changed. Specifically, the proportion of crystalline phases decreased, whereas the proportion of the amorphous phase increased during sintering. As observed in Figure S2, the disappearance of kaolinite and the emergence of mullite in the post-sintering XRD patterns were prominent. Kaolinite and muscovite in the raw materials decomposed and transformed into other mineral phases during sintering, with a significant increase in mullite concentration. Clay minerals such as kaolinite and muscovite contain structural water within their crystal lattices, which is removed upon heating, leading to the collapse of the crystal structure. Kaolinite and muscovite undergo phase transformations with a continuous increase in temperature, ultimately converting into mullite.

### 3.2. Thermogravimetric Analysis

The TG-DSC analysis results of YX, RX, and SL are presented in Figure S3. Figure S3 shows the TG curves and their derivative curves, as well as the DSC curves and their derivative (DDSC) curves during the sintering process from 30 °C to 1200 °C. The overall mass losses for YX, RX, and SL were 5.17%, 6.45%, and 6.26%, respectively. The sintering process can be divided into three main stages, each corresponding to distinct physical and chemical changes.

At temperatures below 200 °C, two stages of mass loss were observed. This weight loss was primarily attributed to the removal of adsorbed and bound water from the clay materials [15]. The mass loss of RX at temperatures below 200 °C (2.27%) differed from that of the other two materials, mainly reflecting a lower loss of bound and adsorbed water in this temperature range. However, RX exhibited a relatively higher loss of adsorbed water. This could be attributed to its higher concentration of kaolinite, a clay mineral with strong water absorption capacity, in its mineral composition. At temperatures between 400 °C and 600 °C, combining the XRD phase analysis of the different materials, the mass loss in this stage was primarily associated with the decomposition of carbonates and the removal of structural hydroxyl groups [16]. The mass loss of SL in the 400–600 °C range was 5.13%, slightly higher than that of the other two samples. Hydroxyl groups are mainly associated with clay and hydroxide minerals; clay minerals such as kaolinite and muscovite lose structural water within this temperature range. Rietveld refinement of the XRD data for RX indicated a quartz concentration of 36.9%. This was significantly higher than that of the other two materials, suggesting a higher degree of surface silicification in RX particles compared with the other ceramic particles. At temperatures above 1000 °C, new crystalline phases were formed during the sintering process [17]. The TG curves of the three materials displayed similar overall trends, despite subtle differences, mainly due to the variations in phase composition.

### 3.3. Analysis of Zisha Porous Structure

**Figure 1:**
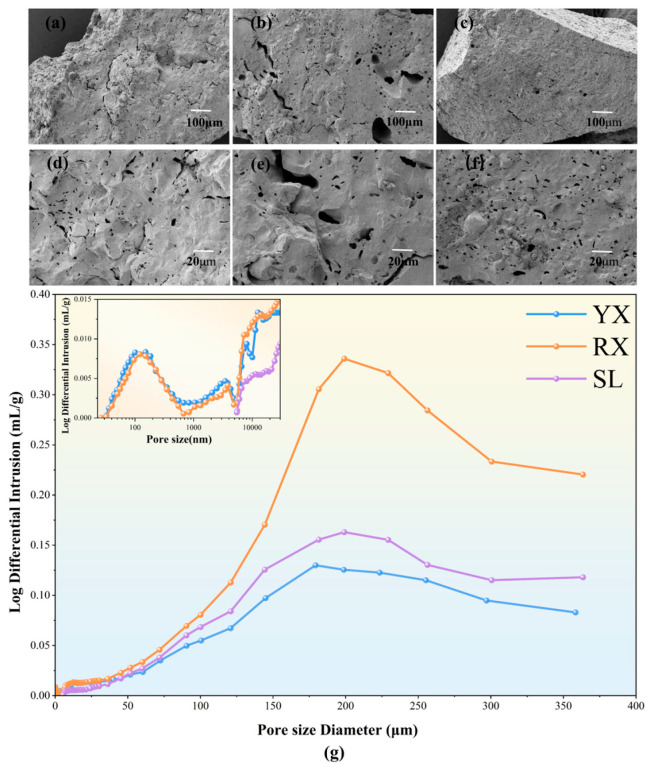
SEM images of YX, RX, and SL particles. (**a**–**c**) 120× magnification for YX, RX, and SL, respectively; (**d**–**f**) 500× magnification for YX, RX, and SL, respectively; (**g**) Pore size distribution of YX, RX, and SL particles measured by MIP.

The surface morphology and the pore size distribution of the three ceramic particles were characterized using scanning electron microscopy and mercury intrusion porosimetry (MIP). As shown in Figure 1, the surface pores of YX, RX, and SL particles were mainly in the micrometer scale. RX particles exhibited distinct large micron-sized pores on the surface, with a nonuniform pore size distribution, which is reflected in the pore size distribution curves shown in Figure 1g. In contrast, the surface pores of YX and SL particles were relatively uniform and smaller in size compared with those of RX particles, which was consistent with the pore size distribution results obtained from MIP.

As shown in Figure 1g, RX particles exhibited larger peak heights and areas. A larger pore peak area indicated a higher proportion of macropore volume, suggesting that RX particles had a higher pore density within the 150–300 μm range. In contrast, the pore distributions of YX and SL particles were more uniform, with YX particles exhibiting the most homogeneous pore distribution, a narrower pore size range, and a more regular microstructure. Further examination of nanoscale pores within 0–1000 nm revealed that YX and RX particles displayed similar MIP pore size distribution profiles, indicating comparable micropore and mesopore concentrations. The abundant microporous structure endows Zisha with a high specific surface area, providing a key advantage during Baijiu aging. Based on previous studies, micropores can efficiently capture pungent compounds such as higher alcohols and free acids, promote ester hydrolysis, and facilitate the volatilization of low-boiling compounds, thereby reducing off-flavors in the liquor. Also, micropores help regulate the micro-oxygen environment, supporting moderate oxidation to generate harmonizing aroma compounds such as aldehydes and pyrazines, thereby enhancing overall flavor balance. In contrast, SL particles exhibited no obvious pore size distribution within this nanoscale range, indicating a deficiency of 0–1000 nm nanopores and, consequently, a weaker adsorption capacity. The MIP pore size distributions were consistent with the pore morphologies observed in SEM images.

The porosity characteristics of YX, RX, and SL, as measured by MIP, are summarized in Table 3. The chemical composition analysis revealed that the differences in pore structure among YX, RX, and SL materials were primarily controlled by their flux concentration and Si/Al ratio. During sintering, Fe_2_O_3_, alkali metal oxides, and alkaline earth metal oxides acted as effective fluxing agents, promoting the formation of a substantial amount of liquid phase. This liquid phase infiltrated the pores, filling numerous voids and resulting in a denser ceramic body [18]. The relatively high iron concentration and low Si/Al ratio in YX material resulted in a moderate amount of liquid phase with high viscosity. This favored the formation and retention of abundant micropores, ultimately leading to a larger total pore area. The high iron concentration, combined with a relatively higher Si/Al ratio in RX material, produced a larger amount of liquid phase with lower viscosity and higher fluidity, allowing rapid filling of small pores and promoting coalescence and coarsening of gas pores. As a result, the pore volume was high, but the average pore size increased. In SL material, despite low iron concentration, the higher concentration of alkali and alkaline earth metal oxides, together with a low Si/Al ratio, generated a substantial amount of highly viscous liquid phase. This led to the merging of small pores into a few large pores, which significantly reduced the total pore area and produced coarser pore sizes. These differences in chemical composition influenced the characteristics of the liquid phase during sintering, ultimately determining the pore distribution of the ceramic particles.

### 3.4. TPR and XPS Analysis

The reducibility of ceramics is a key characteristic for catalyzing Baijiu aging. TPR is an important method for investigating the reduction behavior of materials. The reduction peak temperature and peak area can be obtained by monitoring H_2_ consumption as a function of temperature. As shown in Table 1, the main reducible element in the materials was Fe. The stepwise reduction of Fe at 396 °C, 492 °C, and 679 °C corresponded to the sequential transformation of Fe_2_O_3_ → Fe_3_O_4_ → FeO → Fe [19,20]. Generally, catalysts with lower reduction peak temperatures and higher H_2_ consumption exhibit stronger low-temperature redox performance [21]. The reduction curves are shown in Figure S4, and the quantitative data are summarized in Table S2. Combined TPR and XPS results indicated significant differences in the reducibility and oxidation states of Fe among YX, RX, and SL, confirming the reducibility and catalytic activity of the iron in the ceramic materials.

Figure S5a presents the XPS survey spectra of the surface, whereas Figure S5b shows the XPS survey spectra after 20 nm etching. The three materials primarily contained O (1s, ~530 eV), Si (2p, ~102 eV), Al (2p, ~74 eV), and Fe (2p, ~710 eV), with similar peak intensities. However, the Fe peak of YX was relatively stronger, indicating a higher surface Fe enrichment, consistent with XRF spectroscopy results. Compared with the XPS survey spectra after etching (Figure S5b), the Fe peak intensity displayed slight changes, whereas the O, Si, and Al peak intensities remained nearly unchanged. Peaks around ~290 eV exhibited significant changes before and after etching, corresponding mainly to the characteristic C 1s peak. YX, RX, and SL displayed obvious intensity variations in the C 1s peak around ~290 eV, indicating the presence of surface carbon contaminants or organic residues. This might be attributed to hydrocarbons (C–C/C–H, ~284.6 eV) or oxygen-containing functional groups adsorbed from air exposure. Compared with RX and SL, YX showed the largest C 1s peak intensity change, suggesting that its surface pore structure was more prone to adsorbing organic species.

Figure S5c,d show the high-resolution Fe 2p spectra of the surface and after 20 nm etching, respectively. In the Fe 2p spectra, the peaks were deconvoluted into Fe^3+^ (Fe 2p^3/2^ ~711 eV), Fe^2+^ (~709 eV), and minor satellite peaks. The fitted XPS data are summarized in Table S3. The surface spectra (Figure S5c) showed that RX exhibited an approximately equal proportion of Fe^3+^ and Fe^2+^, whereas SL displayed a stronger Fe^2+^ peak, possibly accompanied by a small amount of low-valence Fe (~707 eV). After etching (Figure S5d), the proportion of Fe^2+^ increased in all three ceramic materials, indicating that the internal Fe was more inclined toward the reduced state. Since the three materials underwent the same sintering process, the proportion of divalent iron in their structures was generally similar. During Baijiu aging, divalent iron acts as a strong reducing agent. It is capable of activating dissolved oxygen or trace hydrogen peroxide in the liquor, thereby initiating Fenton-like reactions [22]. This reaction provides a moderate oxidative environment during aging, thereby promoting the oxidation of alcohols to aldehydes and ketones (e.g., phenylacetone and heptanal) and facilitating the transformation of Maillard intermediates into pyrazine compounds.

### 3.5. Analysis of Liquor Color Changes

As shown in Figure S6a, the KB (blank) sample remained transparent with no noticeable color change, thus maintaining a clear appearance. The liquor samples stored in different materials exhibited distinct color changes, which did not follow a simple progression from light yellow to deep yellow but displayed certain fluctuations. The UV–vis absorption spectra of the Baijiu samples are shown in Figure S6b; samples with darker color exhibited higher absorbance. The absorbance gradually decreased with an increase in wavelength, consistent with the typical absorption characteristics of yellow compounds. We further investigated the cause of liquor yellowing by adding ascorbic acid to the yellow-colored samples as a reducing agent to convert Fe^3+^ into Fe^2+^. Subsequently, the yellow color disappeared (Figure S6d), indicating that the primary cause of yellowing in Baijiu was the presence of trivalent iron.

**Figure 2:**
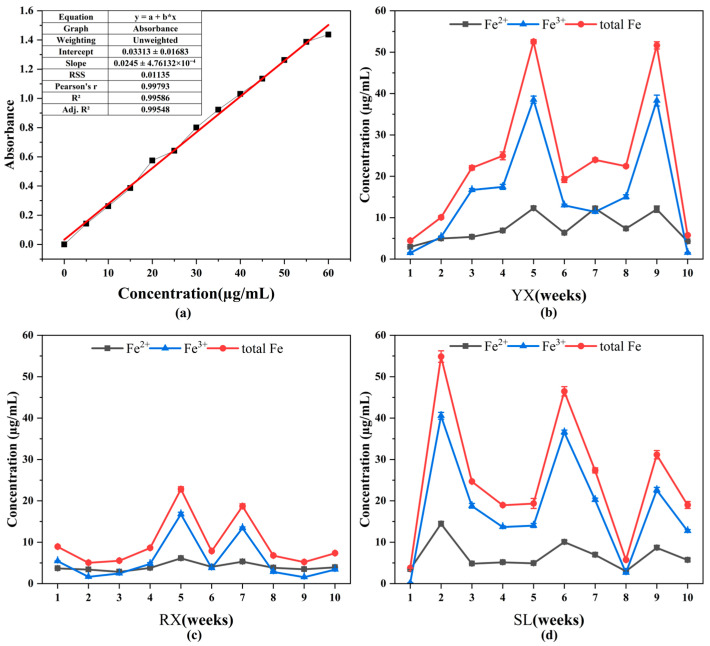
(**a**–**d**) Fe Valence State and Concentration Variations in Baijiu.

The concentrations of Fe^2+^ and Fe^3+^ ions in the samples were measured using a spectrophotometer to verify the iron concentration and its oxidation states in Baijiu. Fe^2+^ ions react with o-phenanthroline to form an orange-red complex. The determination of Fe^3+^ requires reduction to Fe^2+^ using ascorbic acid, followed by color development, thereby allowing the measurement of total iron concentration after reduction. The Fe^3+^ concentration was calculated by subtracting the Fe^2+^ concentration from the total iron concentration. Standard curves were established by preparing solutions with varying iron concentrations and performing the colorimetric reaction, as shown in Figure S6c. The absorbance was measured at 510 nm using a 1 cm cuvette and the blank sample as a reference, yielding the regression equation Y = 0.03313X + 0.0245 with a correlation coefficient R^2^ > 0.995. After reduction with ascorbic acid, Baijiu samples were treated with o-phenanthroline. Also, the color intensity corresponded to the yellow degree of the samples, further confirming the association of the color changes in Baijiu with iron concentration. The concentrations of Fe^2+^, Fe^3+^, and total iron ions in the samples were calculated by measuring the absorbance of different samples and applying the regression equation Y = 0.03313X + 0.0245, as shown in Figure 2.

The iron concentration and its oxidation states in Baijiu exhibited significant differences when stored in different materials. As shown in Figure 2, the iron concentration in YX and SL samples reached up to 57 µg/mL, whereas the RX samples exhibited relatively lower iron concentrations. The iron concentration in Baijiu displayed fluctuating changes during storage in different ceramics, indicating a metal ion cycling effect (MICE). MICE suggested that the metal elements in ceramic particles did not undergo a simple, unidirectional leaching process during Baijiu storage. A similar phenomenon was observed in wine with potassium bitartrate crystallization during storage [23]. Iron in Baijiu coexists in both divalent and trivalent forms, with trivalent iron being predominant. The presence of Fe^2+^ may originate from the ceramic particles themselves or from the reduction of Fe^3+^ by flavor compounds in Baijiu during storage.

The significant color differences observed in Baijiu stored in different materials can be attributed to the variations in iron leaching from the ceramic particles. Iron in Baijiu primarily originates from the leaching of Fe_2_O_3_, which dissolves through reaction with protons: Fe_2_O_3_ + 6H^+^ → 2Fe^3+^ + 3H_2_O. In RX materials, the high SiO_2_ concentration leads to surface silicification. This hinders H^+^ penetration and slows dissolution, resulting in lower iron concentrations in the liquor. In contrast, the porous structure of SL materials generates a capillary effect. This increases the retention time of Baijiu within the pores and prolongs the acid dissolution reaction, thereby promoting efficient leaching of limited iron ions. Additionally, trace elements in SL particles, such as K_2_O (3.32%) and TiO_2_ (1.18%), may synergistically form acidic sites that enhance H^+^ interaction, further accelerating the dissolution process.

The MICE is primarily attributed to the reversible adsorption of iron ions on the material surface. The fate of iron was traced by performing energy-dispersive spectroscopy (EDS) analysis on the ceramic particles before and after Baijiu storage, as shown in Figure S7. Significant changes in surface iron concentration were observed: the Fe concentration on YX, RX, and SL particles increased from 14.22% to 16.83%, from 13.10% to 15.56%, and from 8.28% to 9.30%, respectively. These results indicated that Fe ions in Baijiu could reversibly adsorb onto the particle surfaces. The stored particles were examined at 20,000× magnification and subjected to EDS point scan analysis to further confirm the adsorption behavior (Figure S8). The results clearly demonstrated iron adsorption on the particle surfaces.

### 3.6. Characteristic Flavor Analysis of Baijiu Stored in Zisha

**Figure 3:**
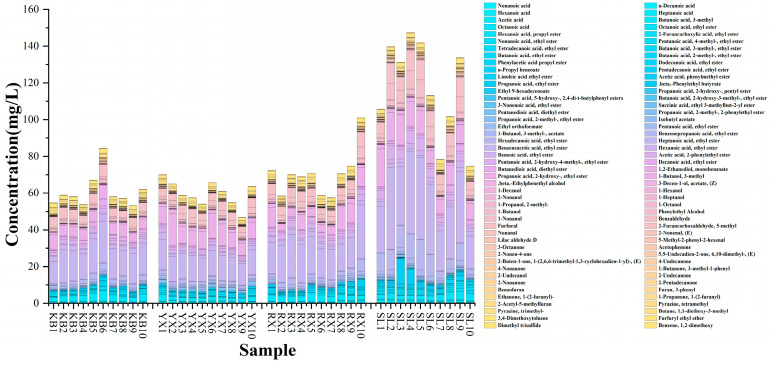
Concentrations of compounds detected in Baijiu during different storage periods.

GC-MS analysis identified 92 trace flavor compounds in Baijiu across the 4 storage conditions, including 7 acids, 42 esters, 12 alcohols, 7 aldehydes, 2 pyrazines, 5 furans, 12 ketones, and 5 other compounds. As shown in Figure 3, the total concentration of volatile compounds in the YX series was significantly lower than that in the SL and RX series. This was likely due to the pore structure, adsorption capacity, and microenvironment of YX particles, which promoted ester hydrolysis and the removal of pungent compounds. In contrast, Baijiu stored in SL particles exhibited the highest total flavor concentration, with a pronounced accumulation of esters, especially higher-molecular-weight species. This likely resulted from the limited presence of nanoscale micropores and weaker adsorption, or from pore structures and microenvironments favoring esterification toward product formation. The RX series displayed intermediate total flavor concentrations, indicating a balanced adsorption–desorption behavior for flavor compounds.

Baijiu stored in YX ceramics exhibited distinctive flavor characteristics, with an overall lower concentration of flavor compounds and a well-defined flavor profile. This storage condition effectively reduced the concentrations of higher alcohols, free acids, and other pungent compounds, resulting in a particularly clean, minimally off-flavored, and remarkably smooth palate. Further analysis revealed relatively higher concentrations of aldehydes and pyrazines in the YX samples, which might be associated with the micropore structure and surface properties of the ceramic particles. These compounds collectively contributed to the harmonious aged aroma of Baijiu. The observed differences may be associated with the abundant micropore distribution and large specific surface area of YX particles, which could enhance physical adsorption and facilitate the volatilization of certain compounds. In addition, the increased release of Fe ions may contribute to oxidative and complexation reactions, potentially promoting the removal of undesirable flavor components.

### 3.7. Analysis of Characteristic Markers in Baijiu Stored in Zisha

**Figure 4:**
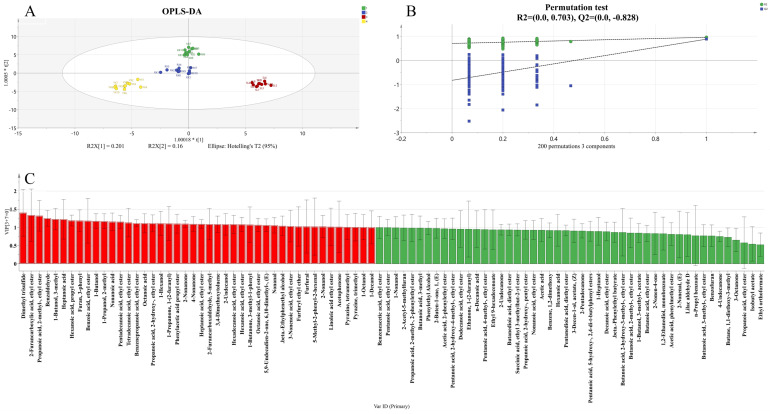
OPLS-DA analysis of Baijiu stored in different materials. The numbers following the letters indicate storage time in weeks. (**A**) OPLS-DA score plot of the samples; (**B**) permutation test performance; (**C**) compounds with VIP > 1 and VIP < 1 are highlighted in red and green, respectively.

Orthogonal partial least squares discriminant analysis (OPLS-DA) is a supervised multivariate statistical method that removes the influence of noncontrolled variables by setting predefined classification variables, thereby extracting deeper data information and quantifying the degree of intergroup differences [5]. It is particularly suitable for classifying datasets with multicollinearity and noisy variables [24]. In this study, OPLS-DA was applied to analyze flavor differences of Baijiu under different storage conditions. The multivariate statistical models were constructed using a completely balanced dataset comprising 40 representative samples (divided into four groups, with 10 time points per group). To minimize analytical variance, the data input for each time point was based on the mean values derived from three independent biological replicates (*n* = 3). Model reliability was evaluated employing a 7-fold cross-validation procedure, with its statistical significance rigorously confirmed via CV-ANOVA (*p* < 0.05). The cumulative R^2^X, R^2^Y, and Q^2^ values for the dataset were 0.835, 0.962, and 0.758, respectively. The score plot (Figure 4A) illustrates significant differences among samples stored in different containers and for different durations. These differences were further explored by conducting a permutation test (*n* = 200) to screen characteristic differences between Baijiu samples and evaluate potential model overfitting. The permutation test randomly rearranged the classification variable (Y) to generate experimental permutations [25]. The results showed that the regression line of Q^2^ points in the permutation plot (black line in Figure 4B) intersected the vertical axis below zero. All permuted Q^2^ points (blue) and R^2^ points (green) on the left were lower than the original values on the right. The R^2^Y intercept was approximately 0.703 and the Q^2^ intercept was around −0.828, indicating a good model fit. Variable importance in projection (VIP) scores revealed the key contributors to classification [26]. The importance of compounds was evaluated using VIP scores, with compounds exhibiting VIP > 1 considered significant [27]. The statistical analysis revealed 44 flavor compounds with VIP > 1 (Figure 4C).

### 3.8. Analysis of Differential Compounds in Baijiu by GC-IMS

**Figure 5:**
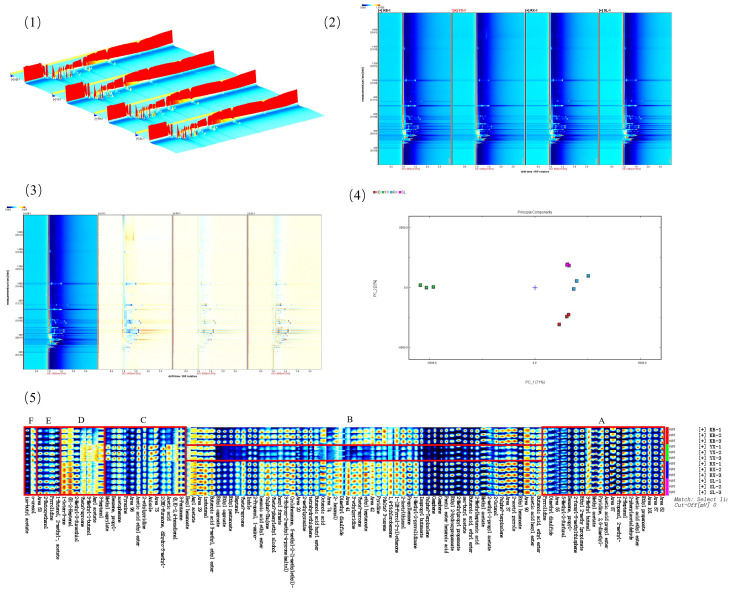
GC-IMS spectra of Baijiu samples: (**1**) 3D top view; (**2**) difference plot; (**3**) Gallery Plot; (**4**) PCA score plot; (**5**) fingerprint map.

GC-IMS analysis was performed on the week-9 storage samples to further investigate the influence of different storage materials on Baijiu flavor. As shown in Figure 5, the difference plot revealed pronounced variations in volatile compounds among the samples. Using KB (blank) as the reference, the YX samples exhibited the greatest deviation, followed by the SL samples. Principal component analysis indicated that the first two principal components accounted for 92.5% of the total variance, demonstrating that these components effectively capture the characteristic differences among the four groups of Baijiu samples.

The flavor compounds in the Baijiu samples were relatively abundant. We categorized the significantly different compounds into six regions (A, B, C, D, E, and F) for further analysis. In Region A, the identified compounds, including ethyl propionate, methyl acetate, and 2-phenylacetaldehyde, were detected in all four samples with relatively high concentrations, laying the foundation for the main aroma notes of Baijiu. In Region B, the targeted compounds, such as ethyl butyrate, ethyl caproate, and hexyl acetate, were present at low concentrations in the YX sample; some even remained undetectable. In Region C, the marked compounds (e.g., pyrazine, acetophenone, and acetic acid) showed higher concentrations in the YX sample. In Region D, compounds including amyl acetate and 3-heptanol were abundant in RX and SL samples, but their concentrations were relatively low in KB and YX samples. The concentration of compounds in Region E (e.g., 2-methyl-1-butyl acetate) increased progressively in the order of KB < YX < RX < SL. In contrast, the concentration of isobutyl acetate in Region F decreased sequentially in the order of KB > YX > RX > SL.

The flavor of Sample KB was dominated by subtle fruit aroma and ethyl acetate. In sharp contrast, Sample YX exhibited remarkable differences: the concentrations of some esters decreased significantly, whereas those of acids and carbonyl compounds increased, endowing it with characteristic acidic, caramel-like, and aged aromas, with a slight reduction in fruit aroma. Sample RX contained moderate concentrations of esters and alcohols, presenting a well-balanced flavor profile with good harmony between fruit aroma and liquor body fragrance. Sample SL had the most abundant species and the highest concentrations of esters; specifically, the concentrations of short-chain esters decreased, whereas the concentrations of long-chain esters and higher alcohols increased, resulting in a smooth and balanced flavor with a rich and stable aroma. In Sample YX, the well-developed nanoscale micropores may have facilitated the selective adsorption of medium-to-high polarity esters (e.g., ethyl caproate and ethyl butyrate), which was consistent with the lower abundance of Region B compounds. Meanwhile, the accumulation of pyrazines fostered dominant, clean, pure acidic, and aged aromas, with the simultaneous retention of high concentrations of acids and carbonyl compounds (e.g., acetic acid, 3-pentanone, and acetophenone), which showed the highest concentrations in Region C.

### 3.9. Analysis of the Correlation Between Flavor Compounds and Metal Ions in Baijiu Stored with Zisha Clay

Freshly brewed Baijiu exhibits relatively low metal concentrations. However, during storage, certain metal ions leach out from the containers, resulting in increased concentrations of Na, K, Mg, Fe, and Mn in the stored Baijiu [28]. When Baijiu comes into contact with clay particles during storage, the concentrations of metal elements, including Na, K, Mg, and Mn, in Baijiu increase significantly, with that of Fe in particular (Figure S9). The ceramic particles are made of fired clay; their main components are SiO_2_ and Al_2_O_3_, along with minor amounts of metal oxides such as Na_2_O, Fe_2_O_3_, CuO, MgO, and TiO_2_ [29]. These metal oxides gradually leached into the Baijiu during storage [30]. In contrast, the concentrations of metal elements such as Fe, Na, Mg, and K in Sample KB and their variations were relatively low and insignificant. The analysis of the percentage changes in metal ions in Baijiu revealed MICE in the liquor system. During Baijiu storage, metal elements not only leached into the liquor matrix but also were reversibly adsorbed onto the surface of ceramic particles, leading to MICE in Baijiu. The leaching behavior of metal elements was closely correlated with the pore structure of ceramic materials. Their leaching rate exhibited a significant correlation with the chemical composition of the materials. Also, the synergistic effects among trace metal elements further promoted metal leaching.

The impact of metal ion release on the characteristic flavor components of Baijiu was further investigated by constructing a Spearman correlation heatmap to analyze the correlations between all detected flavor compounds and metal elements in the samples. The results (Figure S10) showed that benzaldehyde exhibited significant positive correlations with various metal ions including Cu, Mn, Ni, Zn, Fe, K, Mg, and Na (*p* < 0.05); among these, the correlation with Fe ions was the most significant (*p* < 0.001), followed by Mn and Ni ions (*p* < 0.01). Ethyl benzoate displayed significant positive correlations with Mn, Ni, Fe, Mg, and Na ions (*p* < 0.05), with a particularly notable correlation with Fe ions (*p* < 0.01). However, heptanoic acid showed significant negative correlations with Cu and K ions (*p* < 0.05). Propyl caproate exhibited significant negative correlations with Cu, Mn, Ni, and Fe ions (*p* < 0.05); the correlations with Mn and Fe ions were highly significant (*p* < 0.01). Ethyl 2-furoate displayed significant negative correlations with Cu, Mn, Ni, K, and Mg ions (*p* < 0.05), with particularly strong correlations observed for Cu and K ions (*p* < 0.01). Ethyl valerate showed significant negative correlations with Cu, Mn, Ni, and Fe ions (*p* < 0.05), and its negative correlations with Cu, Mn, and Fe ions were particularly prominent (*p* < 0.01). Ethyl caproate exhibited a significant negative correlation with Fe ions (*p* < 0.05). Pentyl 2-hydroxypropionate showed a significant negative correlation with Na ions (*p* < 0.05). Ethyl isobutyrate displayed significant positive correlations with Cu, Mn, and Fe ions (*p* < 0.05), with a particularly strong correlation with Cu ions (*p* < 0.01). Butanol exhibited significant positive correlations with K and Mg ions (*p* < 0.05). 5-Methylfurfural showed a significant negative correlation with K ions (*p* < 0.05). 2-Acetylfuran displayed significant negative correlations with K and Na ions (*p* < 0.05). 2-Propionylfuran exhibited a significant negative correlation with K ions (*p* < 0.05). Other metal ions also displayed significant correlations with most esters and alcohols, suggesting that the metal ions released from the containers may influence the flavor and taste of Baijiu during storage.

**Figure 6:**
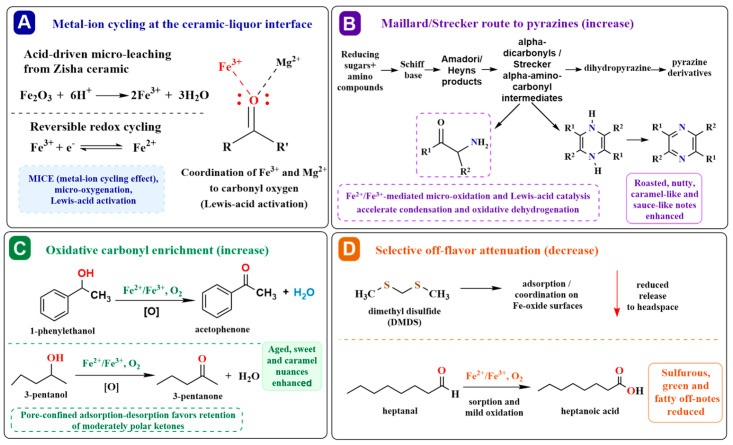
(**A**–**D**) Transformation pathways of flavor compounds.

During Zisha-assisted aging of sauce-flavor Baijiu, the porous aluminosilicate matrix behaves as an active material–flavor interface rather than an inert storage medium. Iron-, magnesium-, manganese-, potassium- and sodium-bearing phases are gradually micro-leached into the acidic ethanol–water system and partially re-adsorbed onto ceramic pore surfaces, generating a dynamic metal-ion cycling effect (MICE) that is proposed to be closely associated with volatile-aroma evolution during Baijiu storage [13,30]. This interfacial process can be described as acid-promoted dissolution of iron oxide to release ferric ions, followed by reversible electron-transfer cycling between ferric and ferrous ions (Figure 6A). The ferrous/ferric redox couple is proposed to sustain a mild micro-oxidative environment through electron transfer and oxygen/peroxide activation, while ferric and magnesium ions act as Lewis-acidic sites capable of coordinating carbonyl or carboxyl oxygen atoms, thereby potentially polarizing carbonyl bonds and presumably facilitating carbonyl-involved transformations [22]. In the Maillard/Strecker branch, residual reducing sugars and amino compounds inherited from high-temperature Daqu and fermentation undergo condensation to Schiff bases, Amadori/Heyns rearrangement, degradation to α-dicarbonyls, and Strecker conversion to α-aminocarbonyl intermediates [31,32]. Under iron-mediated micro-oxygenation and Lewis-acid activation, two α-aminocarbonyl species can condense to dihydropyrazines, followed by oxidative dehydrogenation/aromatization to pyrazine derivatives (Figure 6B), which may contribute to the enrichment of roasted, nutty, caramel-like and sauce-like notes in Zisha-aged samples [33]. In parallel, ferrous/ferric-assisted oxidative dehydrogenation may convert secondary alcohols and aromatic/aliphatic precursors into ketones (Figure 6C), as represented by the oxidation of 1-phenylethanol to acetophenone and 3-pentanol to 3-pentanone. The micro/mesoporous channels further regulate the adsorption–desorption equilibrium of these moderately polar carbonyls, enhancing their persistence in the liquor matrix. Conversely, dimethyl disulfide and heptanal are likely attenuated through selective pore-mediated sorption, reduced headspace release, and coordination/retention on iron-oxide or hydroxyl-rich surfaces (Figure 6D). heptanal may additionally undergo mild aldehyde-to-carboxylic-acid oxidation, or secondary condensation reactions, thereby potentially decreasing sulfurous, green, fatty and aldehydic off-notes [11]. Collectively, the increase in acetophenone, 3-pentanone and pyrazine derivatives, together with the decrease in dimethyl disulfide and heptanal, can be tentatively attributed to the coupled action of metal-ion cycling, iron-mediated micro-oxidative catalysis, Lewis-acid activation, Maillard/Strecker-derived heterocycle formation and pore-confined selective sorption during aging in Zisha ceramics.

### 3.10. Natural Aging Validation and Sensory Assessment

It should be acknowledged that inherent discrepancies exist between the accelerated aging system employed in this study and traditional ceramic vat storage. Driven by a significantly expanded solid–liquid interfacial area, enhanced metal ion leaching, and accelerated mass transfer kinetics, the aging behavior within this system cannot perfectly mirror the protracted reaction dynamics characteristic of industrial storage. Instead, the model was strategically designed as a mechanistic probe to amplify subtle chemical signals and compress the aging timescale. To validate the reliability of the accelerated aging model at a constant temperature of 45 °C, a 9-month long-term natural storage experiment was conducted. This experiment utilized traditional ceramic vat equipped with three types of ceramic particles (YX, RX, and SL), alongside a control group without particles. To monitor the dynamic evolution of the Baijiu’s sensory qualities, samples were collected for sensory evaluation at four critical time points: 1.5, 3, 6, and 9 months. The intensity of the sensory attributes was scored on a 10-point scale (0 = none, 1–3 = weak, 4–6 = moderate, and 7–9 = strong).

**Figure 7:**
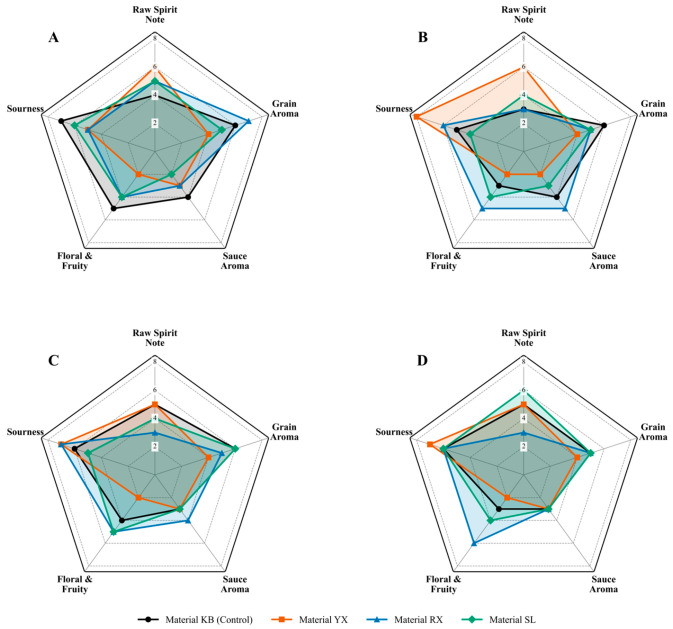
Sensory profiles of sauce-flavor Baijiu under various storage conditions. Panels (**A**–**D**) indicate sensory evaluation scores after 1.5, 3, 6, and 9 months of storage, respectively.

The sensory variations in the sauce-flavor Baijiu across the different storage conditions are illustrated in Figure 7. Regarding the overall sensory profile, over the 9-month storage period, the YX group exhibited a distinct acidic aroma with a slight reduction in fruitiness. The RX group maintained a well-balanced flavor with prominent fruity notes, while the SL group demonstrated a full-bodied and stable aroma profile. The sensory characteristics of the Baijiu naturally aged with the three types of ceramic particles aligned closely with the GC-IMS sensory analysis from the aforementioned accelerated aging experiment, thereby validating the effectiveness of the 45 °C accelerated aging model.

These sensory variations are intrinsically linked to the unique material composition and structural characteristics of the ceramic particles. During the firing process, these particles develop a distinctive porous structure. Concurrently, the abundant metal elements within the particles undergo a slow dissolution process during prolonged soaking in the acidic Baijiu matrix. Consequently, these dissolved metal ions play a pivotal role in the aging process of the Baijiu. Although these sensory trends demonstrate high consistency with the instrumental data, the five-member expert panel in this study primarily served as a preliminary screening. Future large-scale expert evaluations are warranted to further corroborate the conclusions regarding overall sensory harmony.

## 4. Conclusions

This study integrated materials science and food flavor chemistry approaches, progressing from a holistic understanding to multidimensional analysis. It compared differences in chemical composition and physical structure among various Zisha clay materials and explored the correlation between Zisha clay properties and the Baijiu aging process by combining material characterization with Baijiu characterization. The characterization and analysis of different materials and Baijiu samples revealed that the pore structure of Zisha clay was directly related to its chemical composition. Abundant Fe_2_O_3_, alkali metal oxides, and alkaline earth metal oxides in the clay served as effective fluxes, regulating the formation of the pore structure of the ceramic particles. The characteristics of the pore structure were mainly influenced by the flux concentration and Si/Al ratio. During Baijiu storage, the leaching of metal elements was closely associated with the SiO_2_ concentration. The presence of SiO_2_ siliconized the surface of particles, thereby impeding H^+^ penetration and reducing the leaching rate of metal elements. The leaching rate of metal elements was closely associated with the pore structure, whereas the leaching rate of individual metal elements was further impacted by the synergistic effects of other trace metal elements. During Baijiu storage, metal elements not only leached into the liquor matrix but also underwent reversible adsorption onto the surface of ceramic particles, resulting in the MICE of metal elements in Baijiu. The analyses via ICP-MS, GC-MS, GC-IMS, and OPLS-DA demonstrated that Baijiu samples stored in different ceramic particle materials exhibited significant differences in metal element concentrations and volatile flavor compound profiles. The results demonstrated that Baijiu stored with YX Zisha particles exhibited pronounced differences in both metal ion concentration and volatile flavor composition. Specifically, the concentrations of acetophenone, 3-pentanone, and pyrazine derivatives significantly increased. Based on previous studies, such changes may be related to oxidation and other physicochemical interactions mediated by the Zisha matrix, which could contribute to the intensification of roasted and complex sauce-like aroma notes. In contrast, the concentrations of dimethyl disulfide and heptanal were markedly reduced; this observation may be related to the adsorption of sulfur-containing compounds and aldehydes by the ceramic material, which could potentially contribute to improved flavor purity and harmony. These variations were closely associated with the physical structure and chemical composition of the Zisha particles. Metal ions exerted catalytic effects on the formation of volatile flavor compounds in Baijiu, whereas the pore architecture of the ceramic particles regulated flavor release through adsorption–desorption behavior. Sensory evaluation from natural aging in ceramic further validated that, over an extended period, Zisha materials significantly eliminate off-flavors from the Baijiu matrix and enhance overall sensory harmony. These macroscopic sensory profiles exhibited high consistency with the GC-IMS analysis results, thereby demonstrating the immense industrial application potential of Zisha materials in Baijiu aging.

In conclusion, the properties of ceramic materials are crucial during Baijiu storage because they directly influence the sensory quality of Baijiu. Future studies should systematically evaluate the impact of different storage materials on the Baijiu aging process and further investigate the interaction mechanisms between metal ions and flavor compounds during storage. This study provides an important reference for choosing appropriate aging containers and proposes key strategies for further enhancing the development of the unique flavor and sensory characteristics of Baijiu.

## Figures and Tables

**Table 1:** Chemical composition of YX, RX, and SL after sintering at 1200 °C (wt %).

Oxide	SiO_2_	Al_2_O_3_	Fe_2_O_3_	MgO	CaO	Na_2_O	K_2_O	MnO	TiO_2_	P_2_O_5_
YX	59.36	22.42	12.19	0.73	0.14	0.20	3.17	0.03	1.21	0.09
RX	66.88	16.21	12.13	0.64	0.40	0.14	1.87	0.03	1.38	0.05
SL	64.16	22.63	6.74	0.86	0.39	0.13	3.32	0.02	1.18	0.09

**Table 2:** Quantitative Rietveld analysis of YX, RX, and SL before and after sintering.

Sample	Raw YX	Raw RX	Raw SL	YX	RX	SL
Quartz	35.8%	38.2%	37.1%	34.2%	36.9%	35.4%
Kaolinite	15.6%	16.2%	15.8%	0%	0%	0%
Muscovite	33.6%	30.5%	33.2%	0%	0%	0%
Mullite	0%	0%	0%	30.1%	29.2%	28.3%
Hematite	7.3%	6.8%	5.3%	8.2%	7.8%	5.6%
Amorphous Phase	8.7%	8.3%	8.6%	27.5%	26.1%	30.7%

**Table 3:** Porosity characteristics of YX, RX, and SL particles.

Sample	Bulk Density (g/mL)	Pore Volume (mL/g)	Total Pore Area (m^2^/g)	Porosity (%)
YX	2.02	0.09	0.250	17.47
RX	1.95	0.16	0.214	31.51
SL	1.97	0.09	0.040	17.54

## Data Availability

The original contributions presented in this study are included in the article/Supplementary Materials. Further inquiries can be directed to the corresponding authors.

## Supplementary Materials

The following supporting information can be downloaded at: https://www.mdpi.com/article/10.3390/foods15142477/s1. Figure S1. Sintering process. Figure S2. XRD patterns of YX, RX, and SL before and after sintering. Figure S3. TG-DSC analysis of (a) YX, (b) RX, and (c) SL. Figure S4. H_2_-TPR curves of YX, RX, and SL particles. Figure S5. (a) XPS survey spectrum; (b) XPS survey spectrum after 20 nm etching; (c) Fe 2p high-resolution spectrum; (d) Fe 2p high-resolution spectrum after 20 nm. Figure S6. Color changes of Baijiu samples. Figure S7. EDS spectra of YX, RX, and SL particles before and after Baijiu storage: 1–6 correspond to YX, RX, and SL, respectively, pre- and post-storage. Figure S8. EDS spectra of YX, RX, and SL particles after Baijiu storage: 1–3 correspond to YX, RX, and SL, respectively. Figure S9. Percentage Composition of Detected Metal Ions in Samples with Different Storage Times. Figure S10. Correlation Heatmap between Metal Ions and Flavor Compounds. Table S1. Chemical composition of YX, RX, and SL raw materials (wt%). Table S2. Quantitative H_2_-TPR data for YX, RX, and SL particles. Table S3. XPS Fitting Data for Fe in YX, RX, and SL Particles.

## Abbreviations

The following abbreviations are used in this manuscript:

YX	Jiangsu Zisha
RX	Sichuan Zisha
SL	Sichuan ceramic clay
XRF	X-ray Fluorescence Spectroscopy
XRD	X-ray Diffraction
TG-DSC	Thermogravimetric Analysis–Differential Scanning Calorimetry
MIP	Mercury Intrusion Porosimetry
TPR	Temperature-Programmed Reduction
XPS	X-ray Photoelectron Spectroscopy
ICP-MS	Inductively Coupled Plasma Mass Spectrometry
GC-MS	Gas Chromatography–Mass Spectrometry
GC-IMS	Gas Chromatography–Ion Mobility Spectrometry
OPLS-DA	Orthogonal Partial Least Squares Discriminant Analysis
