# Supplementary material for: Zisha Ceramics Modulate Metal-Ion Cycling and Volatile Aroma Evolution During Sauce-Flavor Baijiu Aging
Source: Foods. 2026 Jul 13;15(14):2477. doi: 10.3390/foods15142477 (PMC13408923; doi:10.3390/foods15142477)
Supplement: Supplementary file 1 [file foods-15-02477-s001.zip › foods-4370376-supplementary.pdf]

GC-MS Analysis: Quantitative analysis was conducted using a mixture of two internal standards (2-octanol and 2-acetylpyridine). A 0.5 mL aliquot of the Baijiu sample was transferred into a vial, and 1.5 g of NaCl was added to adjust the ionic strength. The sample was equilibrated at 50 °C for 5 min, followed by solid-phase microextraction (SPME) at 50 °C for 50 min. Subsequently, the SPME fiber was inserted into the GC injection port and desorbed at 230 °C for 5 min in splitless mode. High-purity hydrogen was employed as the carrier gas at a constant flow rate of 1.0 mL/min. The oven temperature program was configured as follows: the initial temperature was held at 45 °C for 3 min, ramped to 150 °C at a rate of 4 °C/min and held for 2 min, increased to 200 °C at 6 °C/min, and finally raised to 230 °C at 10 °C/min, where it was maintained for 10 min. The ion source temperature was set at 230 °C. Mass spectra were acquired in electron impact (EI) mode with an ionization energy of 70 eV, and the scanning mass-to-charge (m/z) range was 35-400 amu.

ICP-MS Analysis: An accurately measured 10 mL aliquot of the Baijiu sample was evaporated to near dryness on a hot plate at 80 °C. Following the addition of 4 mL of nitric acid, the digestion temperature was incrementally increased: first to 120 °C for 30 min, then to 140 °C for 30 min, and finally to 160 °C for 30 min. The remaining nitric acid was then evaporated to near dryness at 100 °C. Ultrapure water (2 mL) was added twice, and the solution was evaporated to near dryness each time. The resulting residue was reconstituted and brought to a final volume of 10 mL in a volumetric flask using 1% nitric acid. Finally, the blank solution, mixed standard solutions, and sample solutions were sequentially injected for ICP-MS determination.

GC-IMS Analysis: The Baijiu sample was diluted 30-fold, and a 1 mL aliquot was transferred into a 20 mL headspace vial. The sample was incubated at 60 °C for 10 min prior to injection. The GC-IMS analysis was performed with a total run time of 30 min. Separation was achieved using an MXT-5 capillary column (15 m×0.53 mm) maintained at a column temperature of 60 °C. Nitrogen (N2) was utilized as both the carrier and drift gas. The IMS detector temperature was set at 45 °C. The automated headspace injection parameters were configured as follows: injection volume, 100 μL; incubation time, 10 min; incubation temperature, 60 °C; syringe temperature, 85 °C; and agitation speed, 500 rpm.

For the volatile profiling via GC-MS, compound identification was achieved with high confidence through a dual-criterion approach: matching mass spectra against the NIST 20 database (similarity score > 800) and cross-verifying

calculated retention indices (RIs) (derived from a C7-C40 n-alkane series) with literature values. The limits of detection (LOD) and quantification (LOQ) for GC-MS were determined at signal-to-noise (S/N) ratios of 3 and 10, respectively. For the elemental analysis via ICP-MS, quantification was based on external multi-point calibration curves ($R^2 > 0.99$), with online internal standards continuously introduced to correct for matrix effects. The LOD and LOQ for ICP-MS were rigorously defined as 3 and 10 times the standard deviation of 11 consecutive procedural blanks.The final quantitative results were reported in mg/L for volatile flavor compounds and μg/L for metal ions.

Table S1. Chemical composition of YX, RX, and SL raw materials (wt %).

Oxide	SiO_2	Al_2O_3	Fe_2O_3	MgO	CaO	Na_2O	K_2O	MnO	TiO_2	P_2O_5
Raw YX	59.54	24.18	10.54	0.79	0.13	0.21	3.10	0.02	1.19	0.07
Raw RX	67.05	18.79	9.56	0.68	0.28	0.14	1.77	0.02	1.21	0.04
Raw SL	62.65	24.77	6.28	1.09	0.45	0.11	3.28	0.01	1.03	0.08

Table S2. Quantitative H_2-TPR data for YX, RX, and SL particles.

Sample	Peak Position （°C）	Peak Area (count)	μmol/g
YX	350.7	13,486	6
	700.6	283,870	136
	828.1	290,496	139
RX	259.7	16,463	8
	547.5	91,576	44
SL	411.9	127,508	61
	671.5	290,862	139

Table S3. XPS Fitting Data for Fe in YX, RX, and SL Particles.

Sample	SurfaceFe^{3+} (%)	SurfaceFe^{2+} (%)	Fe^{3+} after 20 nm etching (%)	Fe^{2+} after 20 nm etching (%)

YX	66.3	33.7	65.1	34.9
RX	66.5	33.5	65.6	34.4
SL	65.6	34.4	64.3	35.7

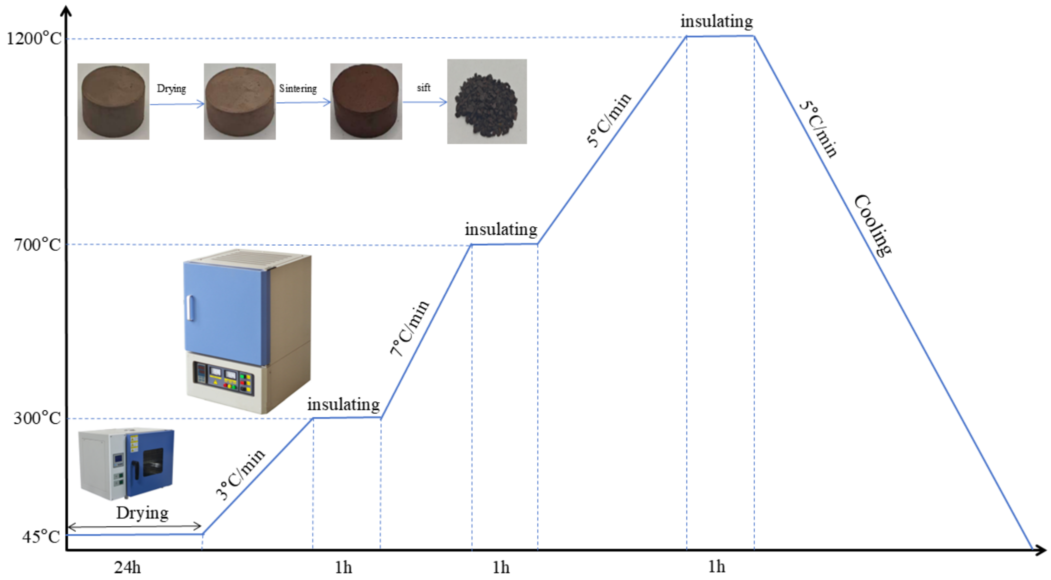

Figure S1. Sintering process.

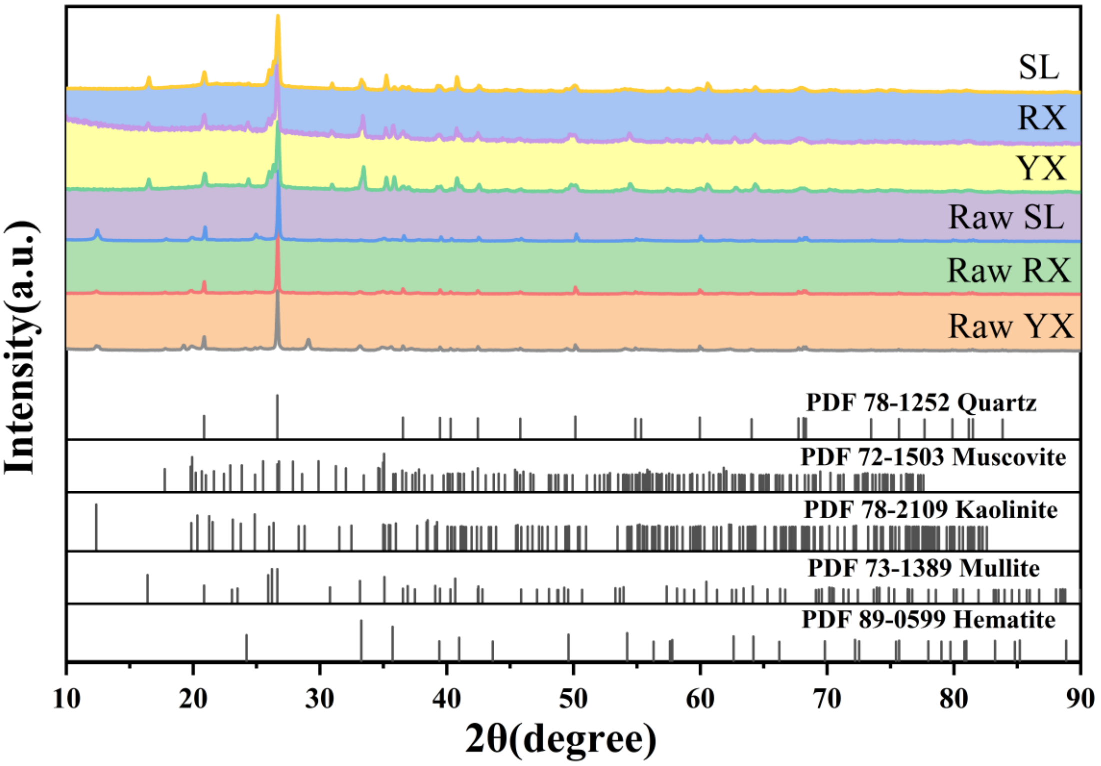

Figure S2. XRD patterns of YX, RX, and SL before and after sintering.

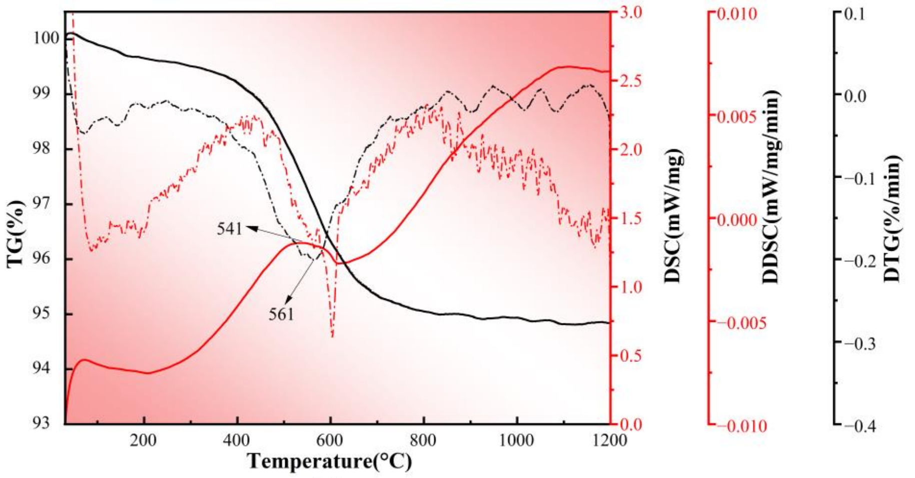

(a)

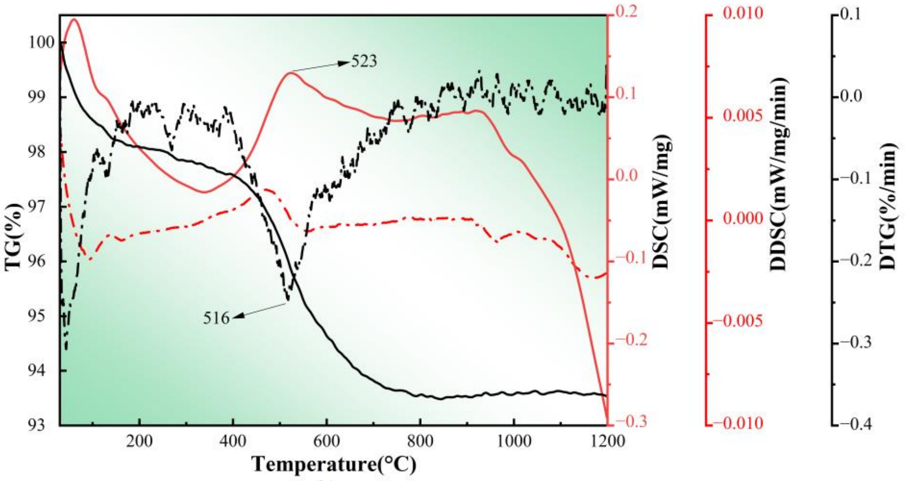

(b)

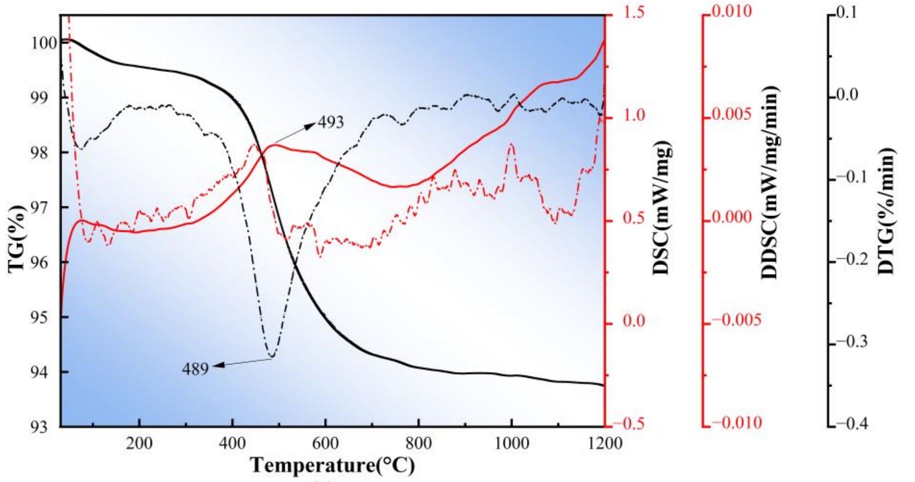

(c)

Figure S3. TG–DSC analysis of (a) YX, (b) RX, and (c) SL.

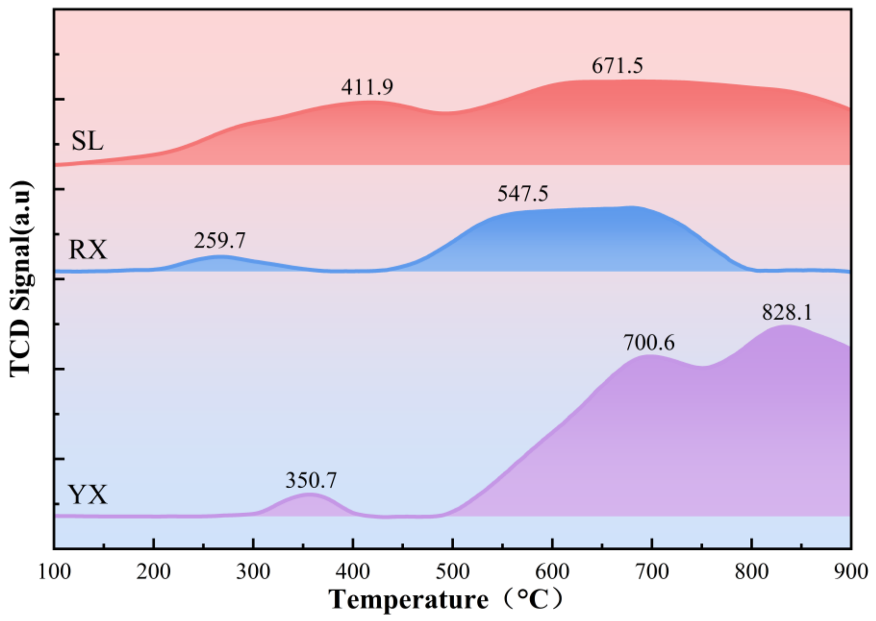

Figure S4. H_2-TPR curves of YX, RX, and SL particles.

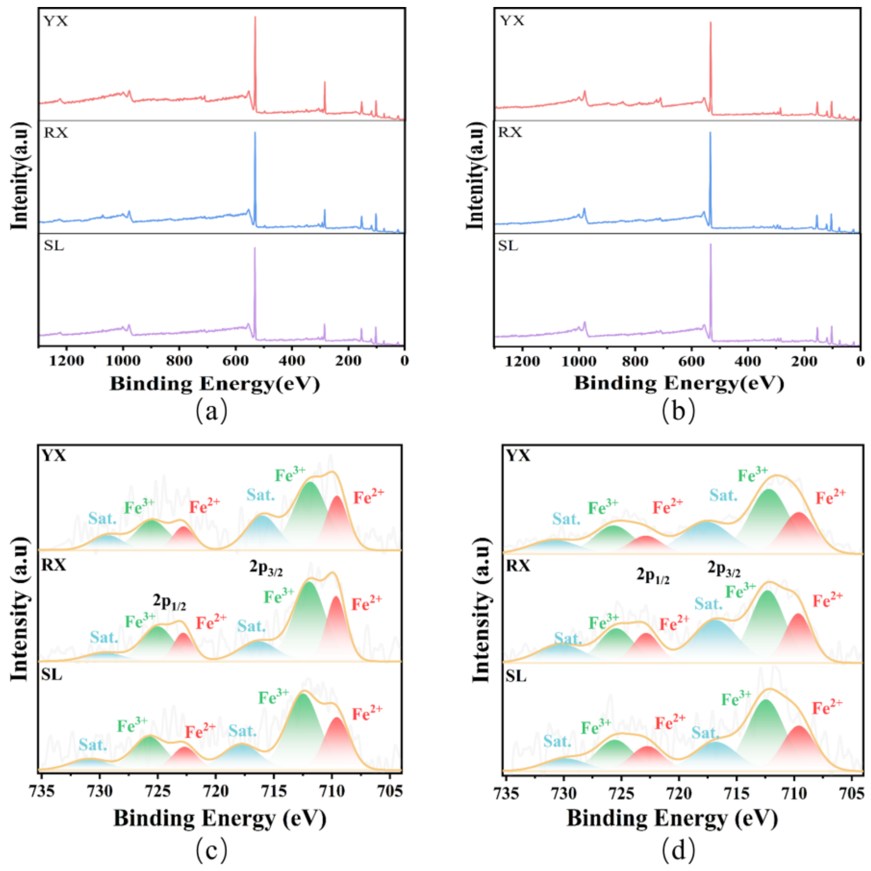

Figure S5. (a) XPS survey spectrum (b) XPS survey spectrum after 20 nm etching (c) Fe 2p high-resolution spectrum (d) Fe 2p high-resolution spectrum after 20 nm etching.

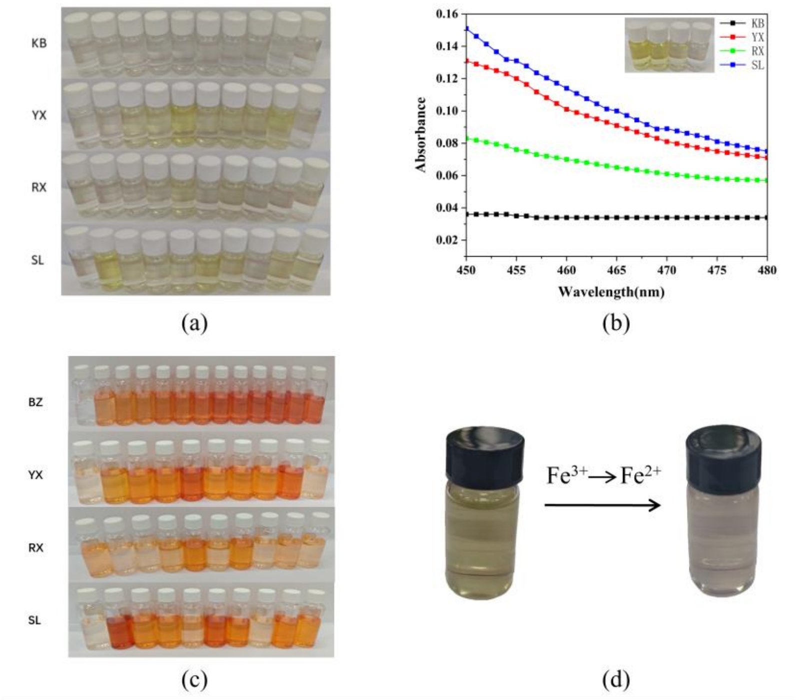

Figure S6. Color changes of Baijiu samples.

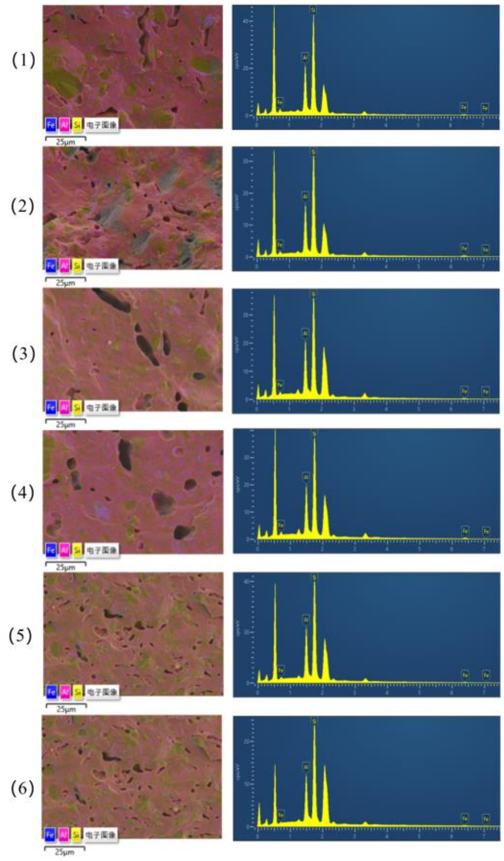

(1)

Element	Wt%	Wt% Sigma	At%
Al	23.11	0.21	25.63
Si	62.66	0.49	66.75
Fe	14.22	0.65	7.62

(2)

Element	Wt%	Wt% Sigma	At%
Al	22.52	0.22	25.33
Si	60.64	0.51	65.52
Fe	16.83	0.68	9.15

(3)

Element	Wt%	Wt% Sigma	At%
Al	24.30	0.30	26.78
Si	62.59	0.69	66.25
Fe	13.10	0.93	6.97

(4)

Element	Wt%	Wt% Sigma	At%
Al	22.77	0.21	25.43
Si	61.68	0.50	66.18
Fe	15.56	0.67	8.40

(5)

Element	Wt%	Wt% Sigma	At%
Al	24.52	0.38	26.34
Si	67.20	0.90	69.36
Fe	8.28	1.19	4.30

(6)

Element	Wt%	Wt% Sigma	At%
Al	22.76	0.36	24.60
Si	67.94	0.96	70.54
Fe	9.30	1.27	4.85

Figure S7. EDS spectra of YX, RX, and SL particles before and after Baijiu storage: 1–6 correspond to YX, RX, and SL, respectively, pre- and post-storage.

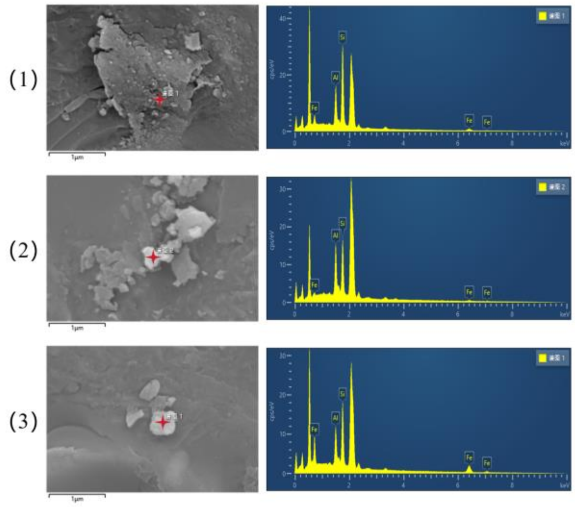

(1)

Element	Wt%	Wt% Sigma	At%
Al	20.02	0.54	24.41
Si	48.96	1.18	57.33
Fe	31.01	1.62	18.26

(2)

Element	Wt%	Wt% Sigma	At%
Al	31.80	1.25	36.02
Si	49.32	1.88	53.65
Fe	18.88	3.02	10.33

(3)

Element	Wt%	Wt% Sigma	At%
Al	11.86	0.34	18.15
Si	22.89	0.55	33.64
Fe	65.24	0.79	48.21

Figure S8. EDS spectra of YX, RX, and SL particles after Baijiu storage: 1–3 correspond to YX, RX, and SL, respectively.

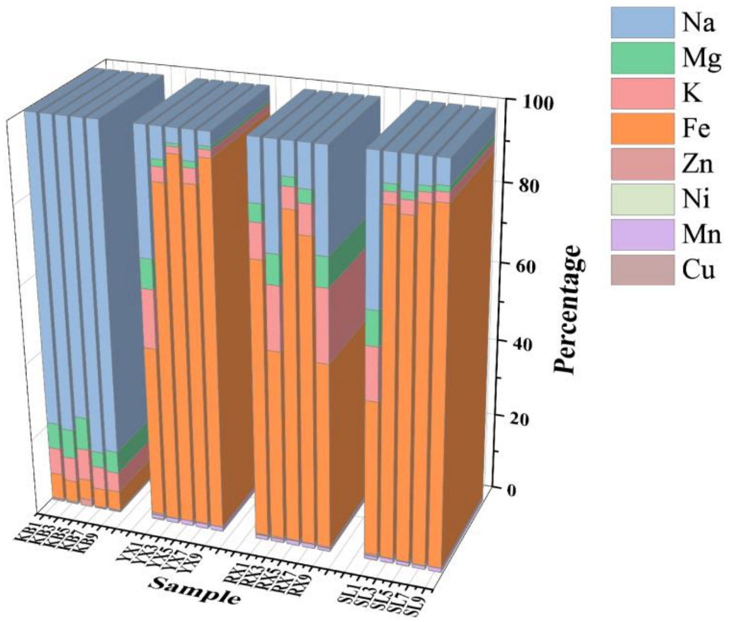

Figure S9. Percentage Composition of Detected Metal Ions in Samples with Different Storage Times.

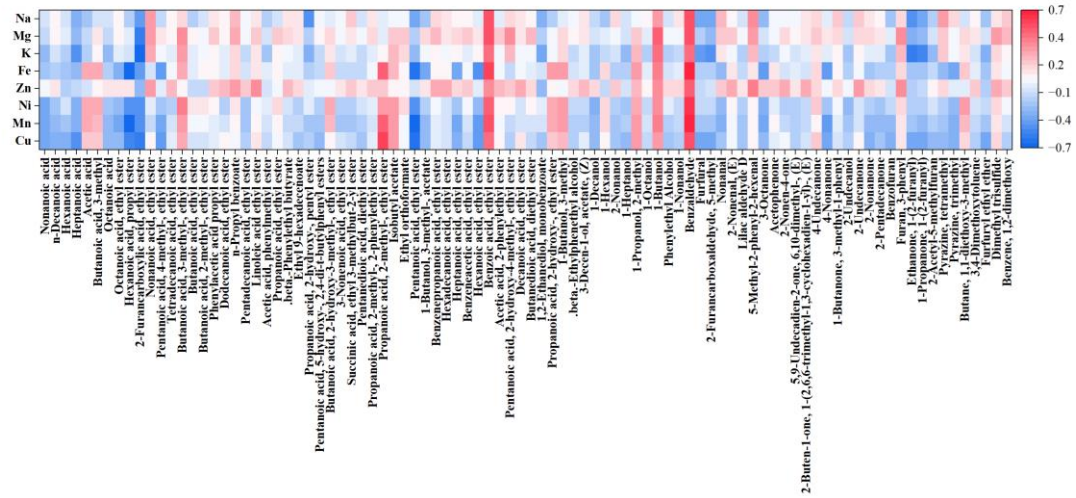

Figure S10. Correlation Heatmap between Metal Ions and Flavor Compounds.
